# Maxillary Overdenture Retained with an Implant Support CAD-CAM Bar: A 4 Years Follow Up Case

**DOI:** 10.2174/1874210601711010247

**Published:** 2017-05-31

**Authors:** Massimo Corsalini, Daniela Di Venere, Gianluca Stefanachi, Giovannino Muci, Andrea Palminteri, Alessandra Laforgia, Francesco Pettini

**Affiliations:** Interdisciplinary Department of Medicine, Dental School, University of Bari, Bari, Italy

**Keywords:** CAD-CAM bar, Overdenture, Prosthetic rehabilitation, Implant rehabilitation, Upper jaw

## Abstract

**Introduction:**

Oral rehabilitation with overdenture on implants of upper jaw must be taken into consideration a variety of anatomical and biomechanical issues. It is possible to provide for rehabilitation with two or more implants, in different positions, solidarizing them with a bar.

**Materials & Methods:**

The present study involved a patient rehabilitated with 4 Xive implants (Friadent GmbH, Mannheim, Germany) solidarized with a titanium bar crafted with CAD-CAM technology for maximal comfort, precision and structural lightness.

**Results & Discussion:**

The follow-up was 54 months, with an implant survival of 100%. Based on our clinical evidence, bars engineered with CAD-CAM technology are promising in terms of precision and comfort despite higher costs.

## INTRODUCTION

According to the definition of Glossary of Prosthodontic Terms [1], “overdenture” is a removable, partial or total, prosthesis supported by one or more residual teeth, roots or implants [2].

The oral rehabilitation of edentulous jaw by combining 4 implants, 2 straight medially and 2 tilted distally, was developed to overcome the anatomic limitations related to bone loss, and where it is difficult to treat the patients without complex augmentative techniques [3, 4]

Cast metal bars have been used in implant supported overdentures as a solution to increase the strength of an acrylic prosthesis and equally distribute load barring forces across the implants. However, the traditional bar for either an overdenture or fixed denture uses cast high noble metal alloys, and because of the increasing cost of gold, complex and time consuming laboratory procedures, commercial laboratories are starting to use the CAD-CAM technology to create the prosthetic framework. Advances in CAD-CAM technology have brought about production techniques, that are faster and more precise, increasing the efficiency in which implant bars are fabricated, and greatly improving predictability upon seating.

Although implant supported overdentures are widely considered an appropriate therapy for edentulous patients, late implant failures have been reported [5], and the most frequent cause is the biomechanical overload of the implants that deteriorates the attachment system [6]. The bar design in itself is biomechanically advantageous [7, 8], and it could increase the success rate of an implant supported prosthesis, especially for maxillary edentulous patients: An implant supported maxillary denture on at least 4 implants and provided with a bar anchorage is a proper treatment option for the edentulous maxilla. Disadvantages of the bar system were also reported, and these were mainly hygiene-related complications due to the difficulty in cleaning the peri-implant zone [9].

The aim of the present study is the critical re-assessment of implant-prosthetic treatment plans in patients with total maxillary edentulia.

The clinical success criteria of the present study comprise [3, 10] optimal aesthetic integration of the prosthetic rehabilitation in the smile exposure and the impact of the frontal group in the facial context; prosthetic retentivity and stability; reinstatement of the masticatory function; easy access to home oral hygiene manoeuvres.

## MATERIALS AND METHODS

**Fig. (1):**
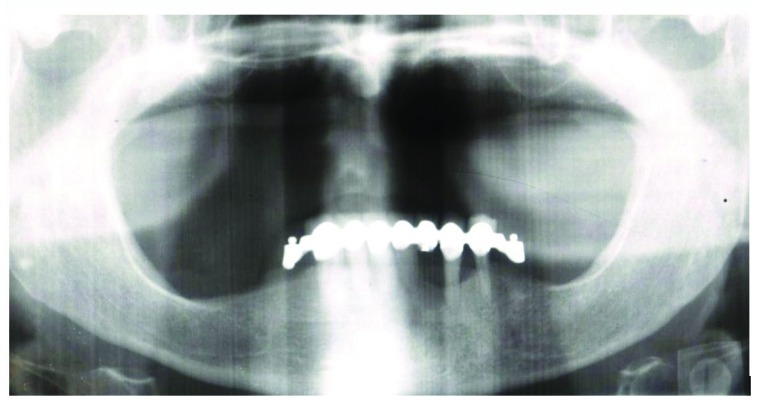
OPT of the selected patient.

A patient 73 years old, female, with maxillary edentulism since three years with significant bone resorption was selected (Fig. **1**), the patient described in the case report has given informed consent for the case report to be published. The patient did not smoke, wasn’t under radiation therapy of head or neck, intravenous bisphosphonate therapy, allergic to restorative materials used.

In the present study Xive^®^ S (Friadent GmbH, Mannheim, Germany) implants were placed with minimal length of 9, 5 mm and maximal length of 11 mm, diameters of 3.8 mm, with cylindrical geometry.

### Surgical Phase

**Fig. (2):**
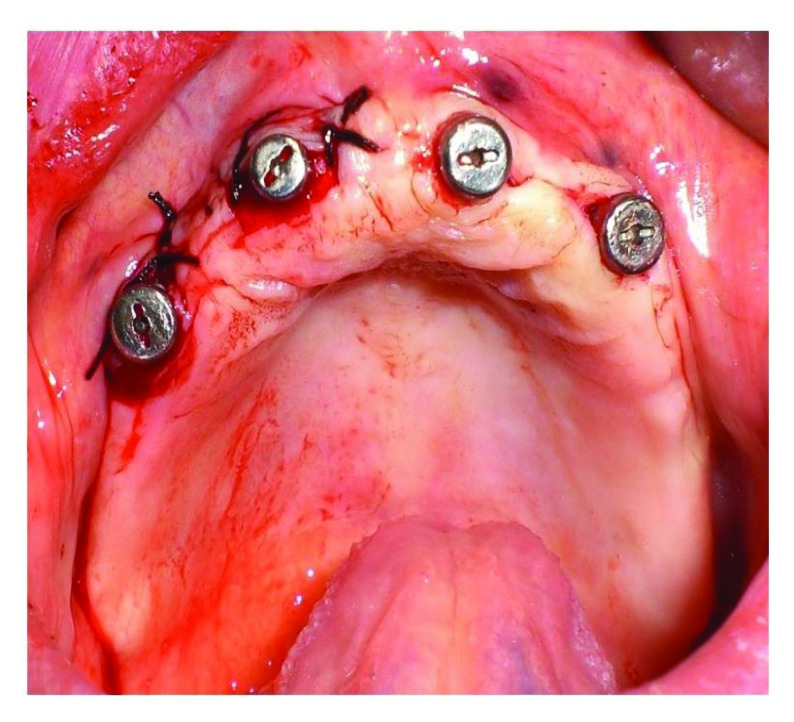
Implants positioned.

Four implants, in just one surgical time and with guided surgery, using a surgical template obtained from a scan with denture in place in patient’s mouth [3], were positioned in the patient: the two distal implants were located in 1.5-2.5 spots, the two central implants in 1.2-2.2 spots (Fig. **2**) [11].

Regarding the importance of symmetry of anchorages, the ideal situation is represented by the presence of two pillars in the canine zones and other two in the pre-molar zones since it enables the realization of a support polygon also avoiding the overdenture rotation axis [3, 10]. Furthermore, great care was given to the control of the axis parallelism. The insertion torque was at least 35 Ncm.

### Prosthetic Phase

**Fig. (3):**
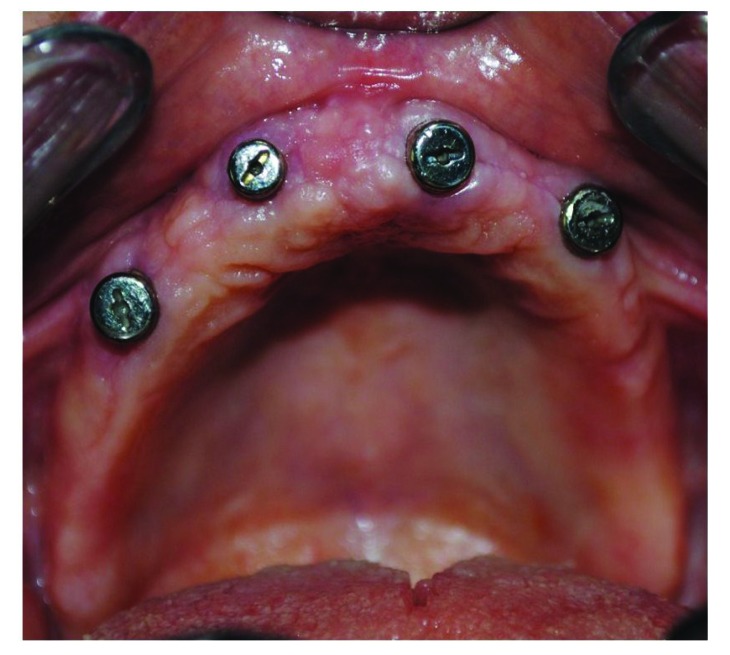
Healing and maturation of tissues completed after six months.

After the osteointegration, healing and maturation of tissues completed after six months (Fig. **3**), we proceeded to the collection of dental print using a polyeter Fig. (**4**) (Impregum DuoSoft H, 3M ESPE, MN, USA).

**Fig. (5):**
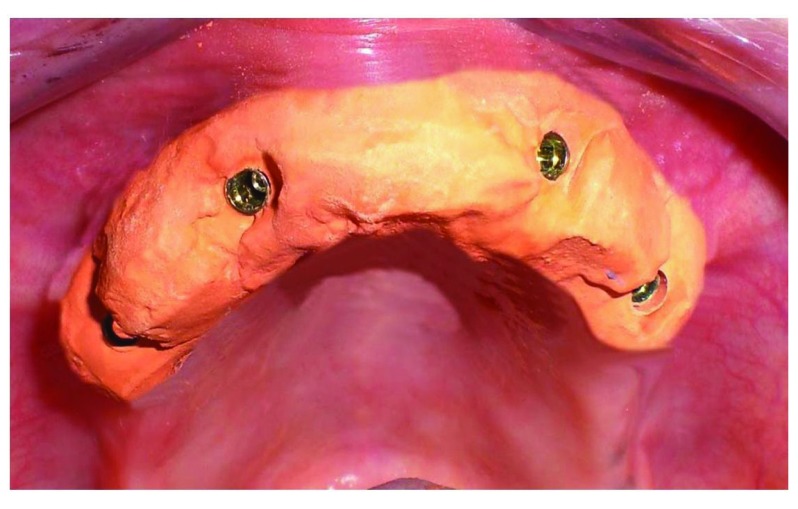
Bar in chalk.

Once the model was crafted, the technician realized a bar in chalk (Fig. **5**): It was screwed together with the stumps directly in the mouth; the absence of fractures is considered a positive result. The precision of the master model was then assessed, it being of fundamental importance as to obtain the passivity of the future structure.

**Fig. (6):**
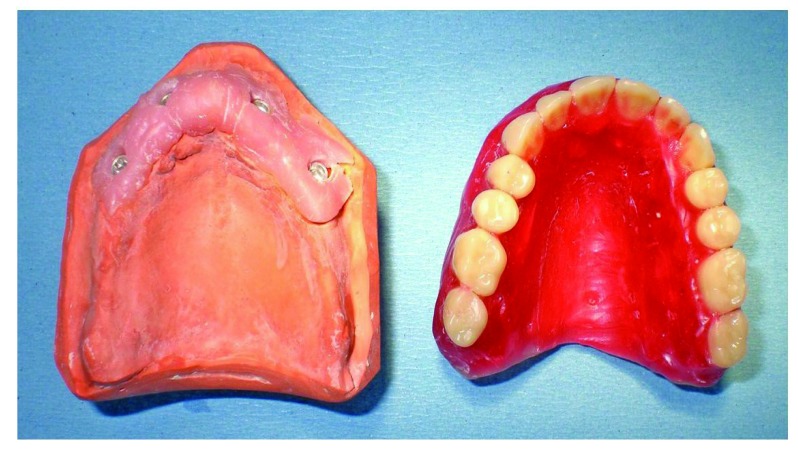
Wax base plate.

Starting from the master model, the technician made a wax base plate (Fig. **6**): An intraoral test was performed so as to verify the vertical dimension, the parallelism in respect to the Camper plan and the bi-pupillary line.

The technician then performed the teeth mounting test.

Once the master model and the wax base were made by means of the teeth mounting test, the data of the models were collected with a dedicated scanner by COMPARTIS ISUS^®^ (Manufacturing Center, E.S. Healthcare N.V. Research Campus 10, 3500 Hasselt, Belgium) and a virtual object was then elaborated in 3D (CAD) [12].

**Fig. (7):**
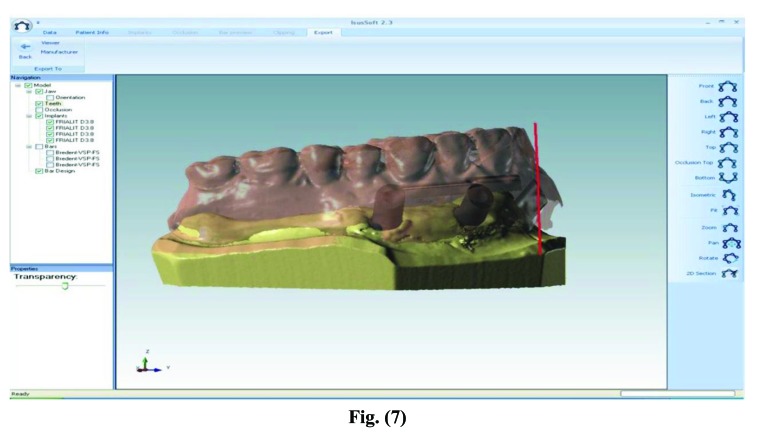


**Fig. (8):**
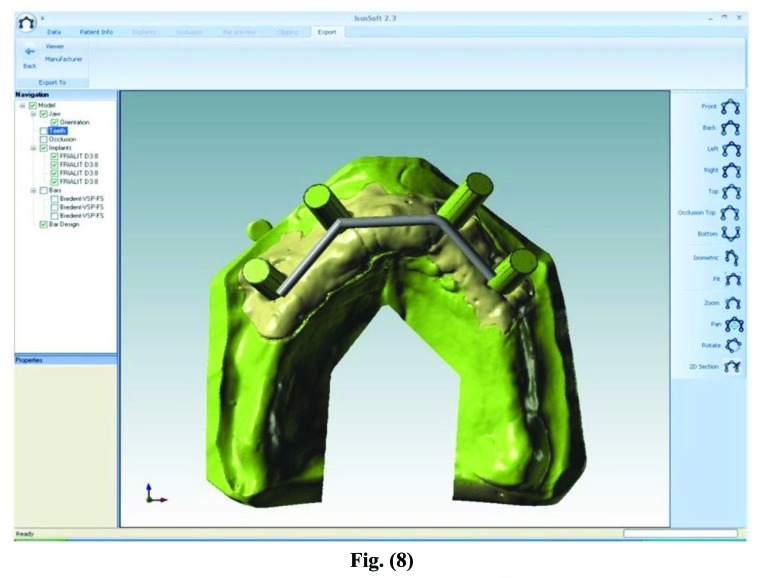


**Fig. (9):**
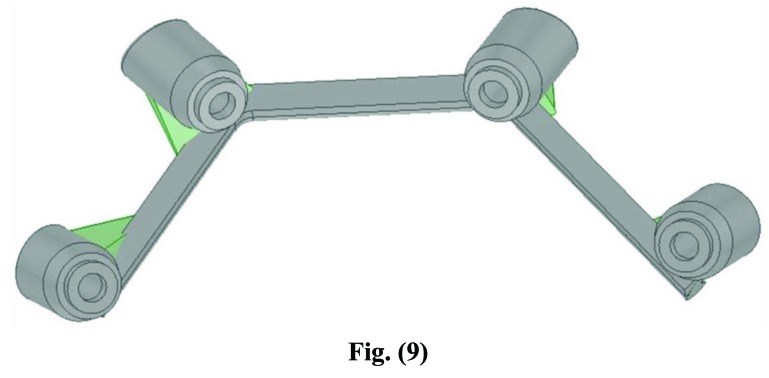
Electronic transmission to the production plant for the CAM milling of the object.

**Fig. (10):**
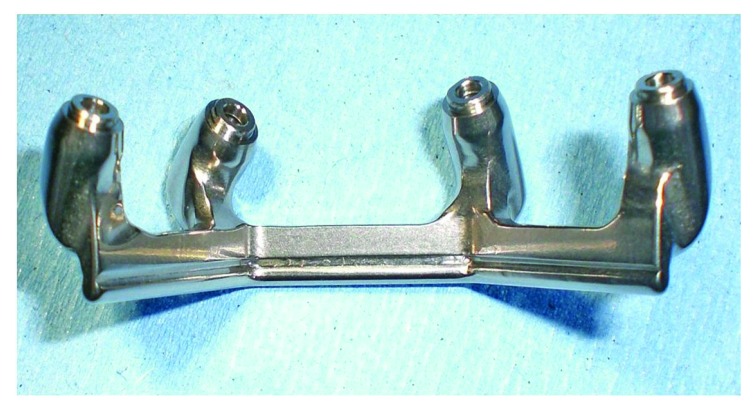
The main connector realized.

After processing, the files were forwarded through electronic transmission to the production plant for the CAM milling of the object according to industrial quality standards (Figs. **7**-**10**).

**Fig. (11):**
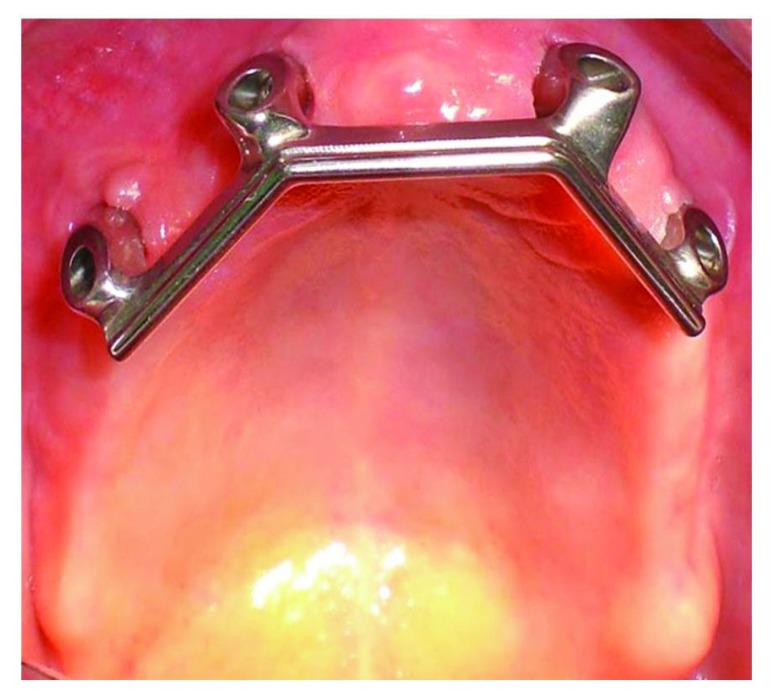
Intraoral test of the main connector.

The CAD-CAM system used was COMPARTIS ISUS^®^ (Manufacturing Center, E.S. Healthcare N.V. Research Campus 10, 3500 Hasselt, Belgium). The intraoral test of the main connector was then performed in order to test the fitting with the bar (Fig. **11**).

## RESULTS AND DISCUSSION

Maxillary implant overdentures with at least 4 splinted implants experienced high implant and prosthetic survival rates [10, 13]; furthermore there was an increased risk of implant loss when 4 or minus implants and non splinted implants were used [14].

**Fig. (12):**
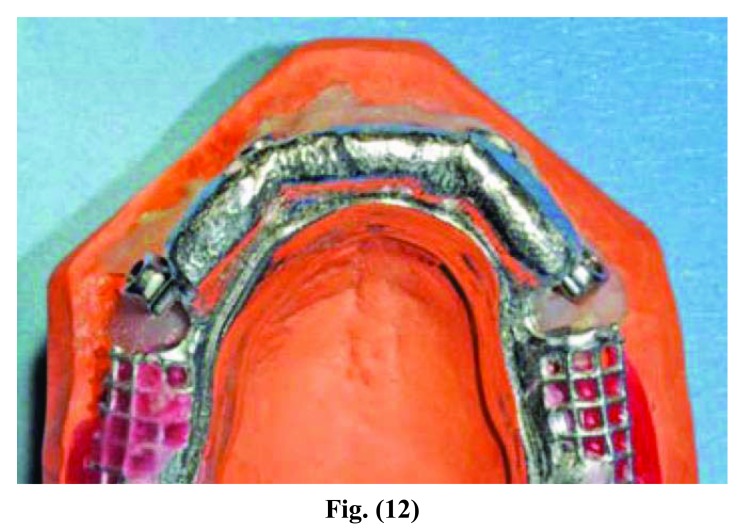


**Fig. (13):**
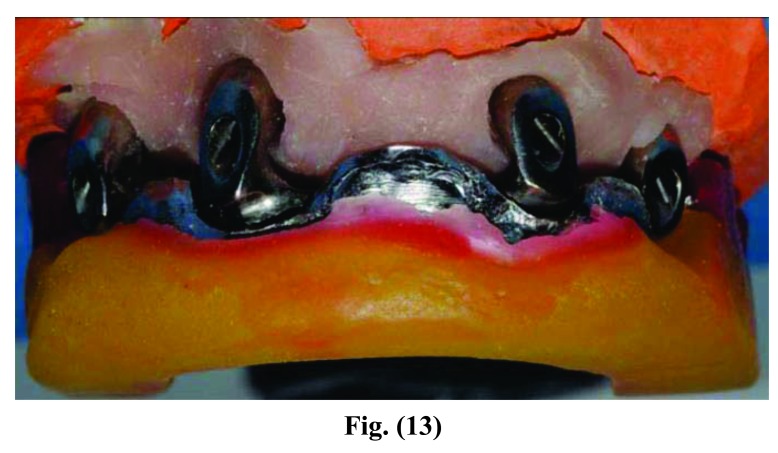


**Fig. (14):**
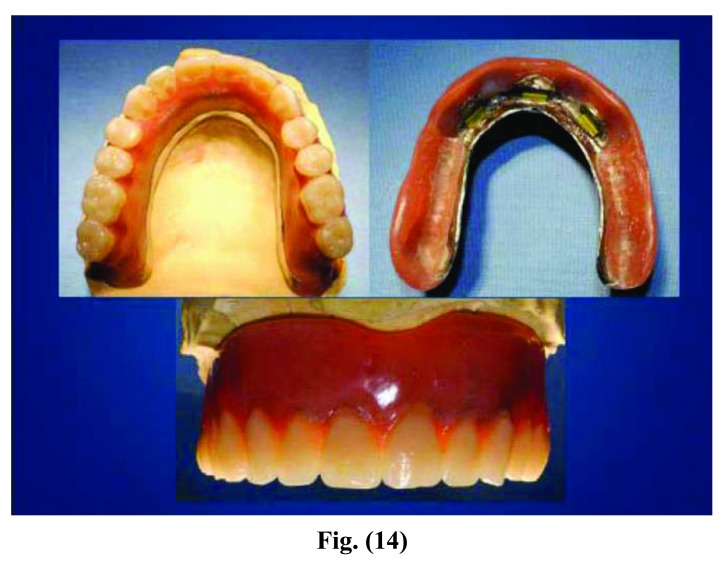


**Fig. (15):**
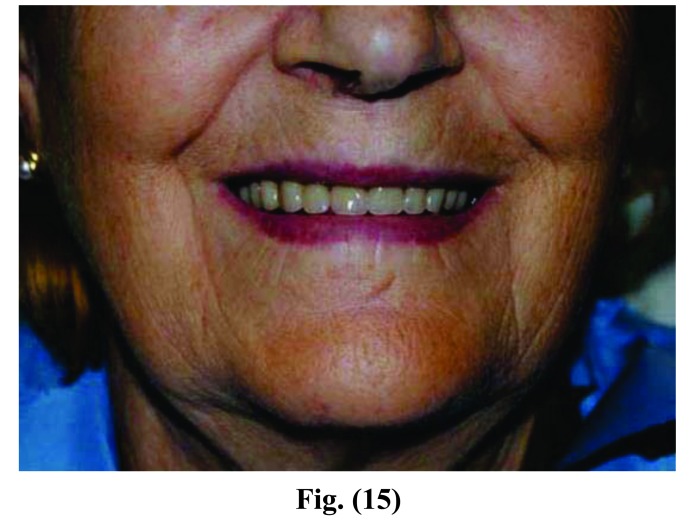
Phases of prosthesis realization.

**Fig. (16):**
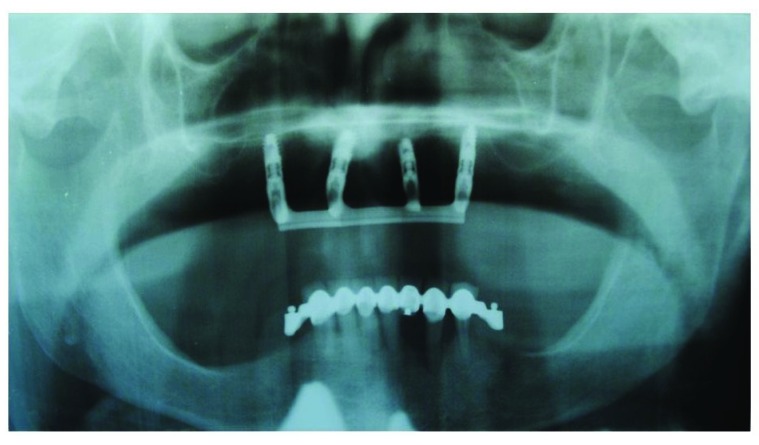
Implant survival after 54 months.

In the present study the implant survival was 100% after 54 months (Fig. **16**). It should be considered that this is a short follow up time but the implant supported rehabilitation has been realized in a patient with severe bone atrophy. Moreover, we achieved a high comfort for the patient thanks to the significant stability of the overdenture and the absence of the prosthetic palate (Figs. **12**-**15**). Patient satisfaction with function and esthetics was assessed by using a scale with ratings 1-10 with 10 = fully satisfied and 1 = not satisfied [3].

Points in favour of the choice of a removable implant support prosthesis are: Simplified, aesthetically profitable rehabilitation even of crests undergoing resorption (it allows to avoid GBR); favourable premises for correct phonetics; compared to mixed-support overdentures bar-sustained overdentures do not display laying nor dislocation and compression of mucosa, minimising the effects of bone resorption typical of removable rehabilitations [3, 10, 15-17].

The use of a bar-sustained overdenture compared to a fixed rehabilitation gives some advantages: a predictable management of the aesthetics; easier home hygiene [18-20]; the total implant support reduces the resorption of the bone crest; avoidance of the resin palate from the prosthetic design granting a higher comfort. In our patient we used a CAD-CAM titanium bar [12] that give us many advantages: tension-less fit; system usable with all implant interfaces; positioning of the structure on implant level (which can be screwed directly to the internal connections of implants) or on abutment; use of only one metal (pure titanium grade 2) [21]; less time consumption and laboratory work; micrometric precision: the producer guarantees discrepancies below 20 μm on the vertical axis, and below 30 μm on the horizontal axis.

## CONCLUSION

The technical reasons for the choice of a CAD-CAM milled bar compared to a bar engineered with conventional techniques are: Solidity [22] (realization from a sole piece and absence of welding); precision [22] (absence of misfit and subsequent passivization at the tightening of the connection screws, and absence of stress on implants and bone-implant interface); lightness (low specific weight of titanium) and possibility to directly insert the milled bar into the internal connection implant.

## Figures and Tables

**Fig. (4):**
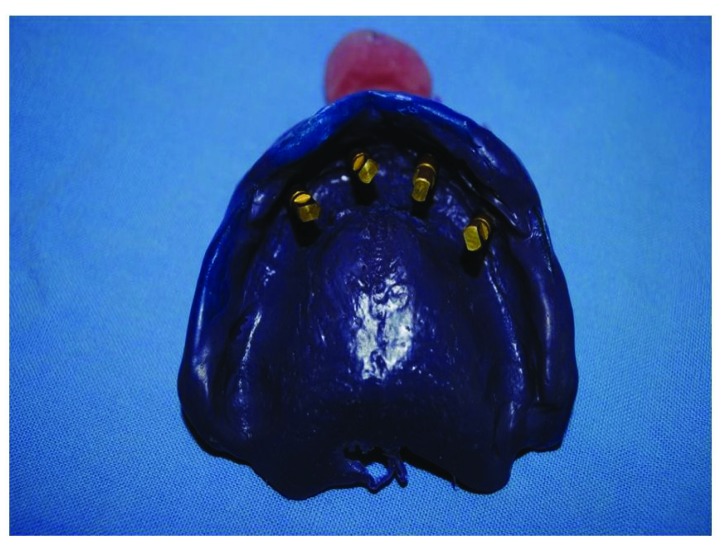
Dental print using a polyeter.

**Table:** 

**Implants position**	**Diameter (mm)**	**Lenght (mm)**
1.5	3.8	9,5
1.2	3.8	9,5
2.2	3.8	9,5
2.5	3.8	11
